# Microbial gas vesicles as nanotechnology tools: exploiting intracellular organelles for translational utility in biotechnology, medicine and the environment

**DOI:** 10.1099/mic.0.000912

**Published:** 2020-04-22

**Authors:** Amy M. Hill, George P. C. Salmond

**Affiliations:** ^1^​ Department of Biochemistry, Tennis Court Road, University of Cambridge, Cambridge, CB2 1QW, UK

**Keywords:** cyanobacterial blooms, gas vesicles, magnetic resonance imaging, nanotechnology, recombinant vaccines

## Abstract

A range of bacteria and archaea produce gas vesicles as a means to facilitate flotation. These gas vesicles have been purified from a number of species and their applications in biotechnology and medicine are reviewed here. *Halobacterium* sp. NRC-1 gas vesicles have been engineered to display antigens from eukaryotic, bacterial and viral pathogens. The ability of these recombinant nanoparticles to generate an immune response has been quantified both *in vitro* and *in vivo*. These gas vesicles, along with those purified from *Anabaena flos-aquae* and *Bacillus megaterium *, have been developed as an acoustic reporter system. This system utilizes the ability of gas vesicles to retain gas within a stable, rigid structure to produce contrast upon exposure to ultrasound. The susceptibility of gas vesicles to collapse when exposed to excess pressure has also been proposed as a biocontrol mechanism to disperse cyanobacterial blooms, providing an environmental function for these structures.

## Introduction

Gas vesicles are hollow, proteinaceous, intracellular organelles that are produced by a range of bacteria and archaea [1]. They were first discovered in cyanobacteria through their tendency to conglomerate in the formation of gas ‘vacuoles’ that refract light [2]. Cyanobacteria that can make gas vesicles include *Anabaena flos-aquae, Planktothrix rubescens* and *Microcystis* species [3]. Gas vesicles have also been identified in a range of heterotrophic bacteria, including *Psychromonas ingrahamii* [4], *Serratia* sp. ATCC 39006 [5], *Bacillus megaterium* [6] and *Streptomyces* sp. CB03234-S [7]. Archaea that produce gas vesicles include halophiles such as *Halobacterium salinarum* [8]*, Haloferax mediterranei* [9] and *Haloquadratum walsbyi* [10, 11].

The genes required for gas vesicle production have been identified in a range of species. While there is homology between some of the core gas vesicle genes, there is also significant variation in the genes required for gas vesicle formation between organisms [1]. In *Halobacterium* species NRC-1 there are two gas vesicle gene clusters present, on plasmids pNRC100 (*gvp1*) and pNRC200 (*gvp2*) [12–15]. Each of these clusters contain 14 genes in 2 divergently transcribed operons [13, 16]. In contrast, the *Anabaena flos-aquae* gas vesicle gene cluster consists of five copies of *gvpA* and homologues of six other gas vesicles genes [17, 18]. In *Serratia* sp. ATCC39006 the gas vesicle cluster is comprised of 19 genes in 2 operons, of which 11 are essential for gas vesicle production [5, 19]. The gas vesicle gene clusters of *Serratia* sp. ATCC39006 and *Bacillus megaterium* have been expressed in *Escherichia coli* and gas vesicle structures have been observed in the heterologous host [5, 6]. The functions of some gas vesicle genes are conserved between species and have been well characterized, such as *gvpA* and *gvpC*, which encode the core structural protein and secondary strengthening protein, respectively. However, there are multiple genes of unknown function in various gas vesicle loci, some of which are predicted to encode minor structural proteins or play a regulatory role [19, 20].

**Fig. 1.**
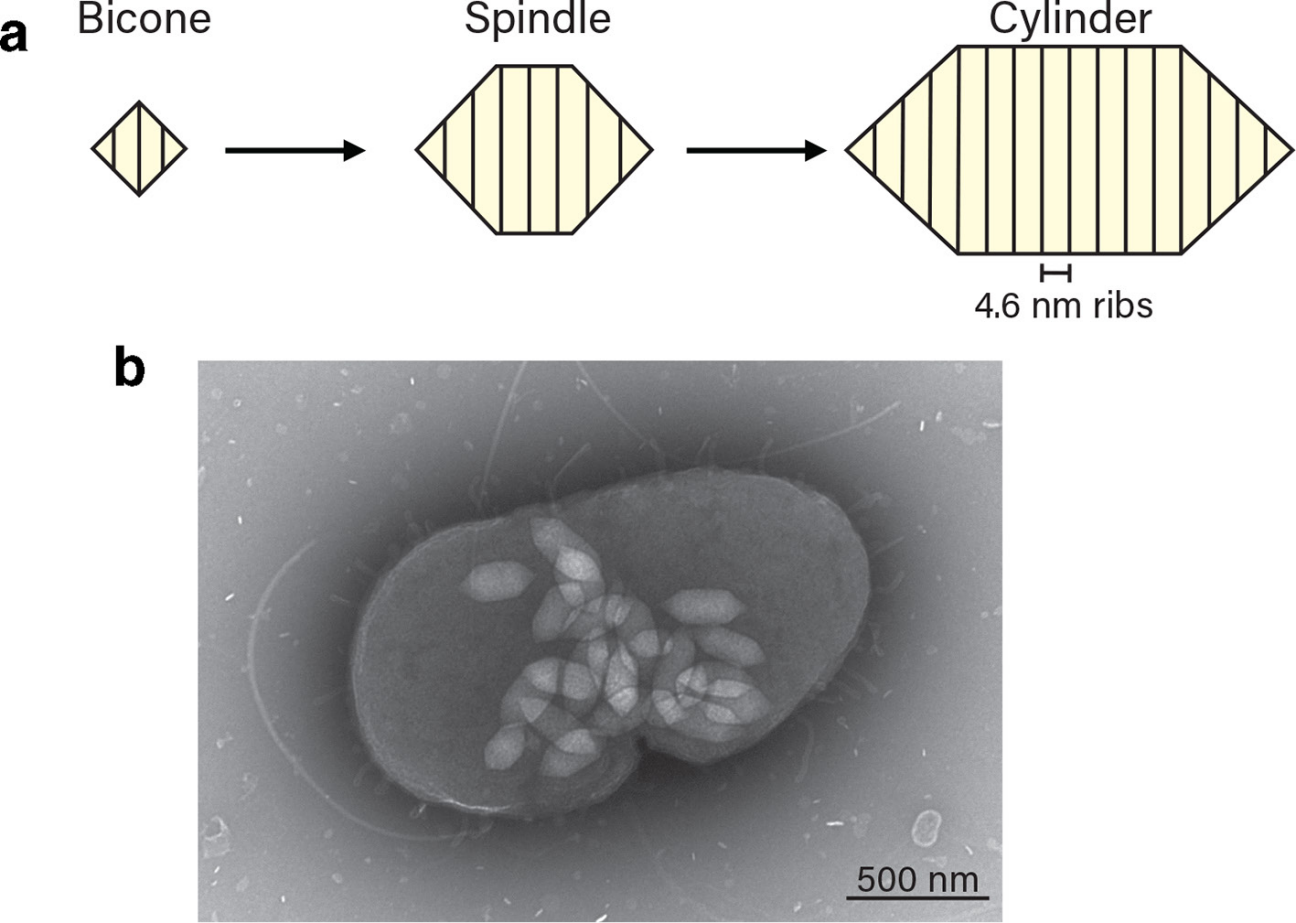
Gas vesicle development and appearance. (a) Gas vesicles develop from small bicone structures into mature spindle/cylindrical structures. (b) Transmission electron micrograph of mature gas vesicles inside a *Serratia* sp. ATCC 39006 cell.

Gas vesicle size and shape varies depending on both the organism and the environmental conditions. Individual gas vesicles tend to be 0.045–0.2 µm wide and 0.1–2 µm long [1]. Vesicles initially form as small bicone structures that then extend to become spindle- or cylindrical-shaped mature gas vesicles (Fig. 1a) [3]. The core gas vesicle protein, GvpA, is a 7–8 kDa protein that assembles into 4.6 nm wide ribs that run perpendicular to the long axis of the vesicle and the 2 nm thick vesicle wall is composed of a single layer of this hydrophobic protein [3, 21]. The wall of the gas vesicle excludes water but allows gas to diffuse freely across it [22, 23]. A secondary gas vesicle protein, GvpC, is often found forming a mesh over the exterior surface of gas vesicles, strengthening the structure [24–26].

Gas vesicles increase the buoyancy of cells and, when present in sufficient quantity, facilitate upward flotation in static water columns [3]. Gas vesicles collapse when exposed to pressure that exceeds the ‘critical collapse pressure’, thereby reducing the buoyancy of the cell [2, 3]. The critical collapse pressure of gas vesicles can be measured using pressure nephelometry and varies depending on the dimensions of the vesicles [27]. Nephelometry has also been used to demonstrate the strengthening effect of the GvpC structural protein on gas vesicles [19]. Narrower gas vesicles tend to be found in organisms that grow in deeper environments and are more resistant to collapse under hydrostatic pressure [27].

Individual gas vesicles can be visualized within cells using transmission electron microscopy (Fig. 1b). Gas vesicles have been purified from various organisms to determine their structure and protein composition [28–32] and there is a growing interest in the use of gas vesicles for biotechnological, medical and ecological applications. For example, gas vesicles are being investigated as antigen delivery vehicles, where promising results have already been observed in a range of systems [33]. Gas vesicles are under investigation as contrast agents for use in magnetic resonance imaging (MRI) and they have been proposed as a target for disrupting cyanobacterial blooms by exploiting ultrasonic collapse of the vesicles [34, 35]. This review will focus on the potential applications of gas vesicles, what has been achieved so far and prospects for future applications.

### The use of gas vesicles in engineering vaccines

Purified gas vesicles engineered to also display an antigen of interest, known as gas vesicle nanoparticles (GVNPs), can offer advantages over other vaccine types, including increased stability, immunogenicity and enhanced uptake across cell membranes [36–38]. Use of GVNPs can also avoid some of the downsides of live-attenuated vaccines, including a lower risk of infection, and they have the therapeutic potential to be given to immunocompromised individuals [33, 37].

Gas vesicles were first proposed as an antigen delivery system nearly 20 years ago and have since been engineered to display antigens from viruses, bacteria and eukaryotes [33, 39]. Most of this work has been performed using purified gas vesicles from the halophilic archaeon, *Halobacterium* sp. NRC-1 [40–45]. *Halobacterium* gas vesicles are an ideal vaccine component due to their biological stability and resistance to chemical or enzymatic degradation, thereby allowing sustained presentation of the epitope of interest [39]. The creation of a range vectors containing the gas vesicle genes enables facile genetic manipulation and production of recombinant GVNPs at low cost [14, 39, 46, 47].

The basic structure of *Halobacterium* gas vesicles involves a highly organized rib structure of GvpA with GvpC located on the outer surface of the vesicle, providing stability and strengthening the structure [48–50]. Modelling studies have suggested that GvpA forms a hydrophobic surface on the inside of the gas vesicle while the external surface is hydrophilic [49, 51]. The acidic tail of GvpC is predicted to be important for protein stability in high-saline conditions and has also been investigated as a region capable of tolerating insertions of antigenic peptides [39]. Previous studies using *Halobacterium* sp. NRC-1 established methods for scaling up the production and purification of gas vesicles [13, 52, 53]. Low-speed centrifugation overnight of lysed *Halobacterium* cells allows the buoyant organelles that rise to the air/liquid interface to be removed and purified [39].

Gas vesicles purified from *Halobacterium* sp. NRC-1 were initially tested without any alterations to determine their immunogenicity before specific alterations to GvpC were investigated [39]. For this study, a gas vesicle-deficient strain of *Halobacterium* sp. NRC-1, SD109, was transformed with the pFL2 vector for gas vesicle purification [39, 54, 55]. Strain SD109 is a spontaneous gas vesicle-negative mutant of *Halobacterium* sp. NRC-1 that has the entire gas vesicle gene cluster deleted [54, 55]. The pFL2 vector is an *E. coli–Halobacterium* shuttle plasmid that contains a 13 kb *gvp* gene fragment, which is sufficient to complement the gas vesicle-negative phenotype of strain SD109 and includes a selectable marker for mevinolin [39, 56]. Gas vesicles purified from *Halobacterium* strain SD109 carrying pFL2 were tested to determine their immunogenicity without alteration and after chemical conjugation of the trinitrophenol (TNP) hapten [39]. Experiments performed in mice showed that wild-type gas vesicles stimulated an immune response but had no negative impact in terms of mouse survival, or any obvious indications of toxicity [39, 42]. Only the TNP conjugated vesicles elicited a TNP-specific antibody response [39]. No external adjuvant was used in conjunction with the gas vesicle preparations, indicating that gas vesicles are capable of acting as both an adjuvant and an antigen delivery system [39]. After demonstrating that gas vesicles could function as an antigen carrier, the ability of GvpC to tolerate insertions while remaining functional and antigenic was tested using an 18 base pair insertion in the C-terminus of the gene [39, 56]. Mice injected with the recombinant gas vesicles displayed a specific immune response against the inserted ESSGTF peptide [39].

This system was then used to express and display simian immunodeficiency virus (SIV) antigens on gas vesicles and monitor the immune response elicited [42]. The insertions ranged in length from 17 to 235 amino acids of the SIV Gag protein, much larger than previously described GvpC insertions [39, 42, 56]. The Gag protein was selected as an antigen as it is a precursor to one of the core structural proteins of SIV [57, 58]. These recombinant SIV–GVNPs were recognized by antibodies produced by SIV-infected monkeys in an enzyme-linked immunosorbent assay (ELISA), indicating that the Gag protein segment adopted an immunologically recognizable conformation on the gas vesicle [42, 50]. Subsequent tests in mice found a long-lived immune response, with antibodies detected 120 days after a booster injection and a rapid IgG response 10 days after a second booster [42].

After the successful expression of the SIV Gag protein on the surface of *Halobacterium* sp. NRC-1 gas vesicles, further work was carried out to express different SIV proteins on recombinant GVNPs and determine their potential use in vaccines [42, 50]. Recombinant SIV–GVNP fusions were made, expressing different SIV proteins, Tat, Rev and Nef1 [50]. The Tat protein is produced early in the virus life cycle and is required for viral replication, and the homologue in human immunodeficiency virus (HIV) has been proposed as a vaccine target [59, 60]. Rev and Nef1 are found in the host cell nucleus and membrane, respectively, and have also been identified as important potential targets for HIV vaccines [61–64]. The Tat/Rev/Nef1 recombinant gas vesicles were confirmed as functional through flotation tests before Western blots were used to show that the SIV–GVNPs were larger than wild-type [50]. Specific anti-Tat/Rev/Nef1 mouse antibodies could recognize the appropriate SIV–GVNPs but not wild-type GVNPs [50]. Anti-GvpC tests were positive for all gas vesicles – wild-type and recombinant [50].

ELISAs were used to determine the immune response of mice injected with one of the three types of SIV–GVNPs and the strongest immune response was shown by GVNPs displaying a portion of the Tat protein [50]. From cultured macrophages, the cytokines produced following exposure to these GNVPs that displayed SIV epitopes were also investigated [65]. Using archived sera samples, the specific antibody isotype was determined for the GVNPs displaying fragments of the Tet, Rev and Nef1 SIV proteins, with IgG1 the predominant isotype [50, 65]. The dominant cytokines released throughout the immunization process were IL-10 and IL-12, which peaked after 12 h for the GVNPs displaying Tat and Rev [65]. For Nef1-GVNPs, the IL10 response peaked after 24 h and was at a much higher level [65]. This work also tracked the time taken for gas vesicles to degrade within macrophages, by visualizing clusters of immunostained GVNPs [65]. Along with Western blots, the study showed that the Tat, Rev and Nef1 proteins were degraded at a much faster rate than the GvpC proteins, which, although partially degraded, still remained at detectable levels within macrophages after 120 h [65].

This recombinant approach was subsequently used to produce GVNPs displaying three different outer-membrane proteins from *Chlamydia trachomatis* [66]. These proteins were: the major outer membrane protein (MOMP), outer membrane complex B (OcmB) and polymorphic outer-membrane protein D (PompD), each of which have been proposed to have roles in the virulence of *C. trachomatis* and have been suggested as potential vaccine candidates [67–69]. Western blots performed using anti-GvpC and anti-* Chlamydia* antibodies, as well as sera from patients with *Chlamydia* infections, were used to detect the presence of MOMP, PompD and OcmB fragments on the surface of the GVNPs [66]. The GVNPs were confirmed to have been taken up by the cells using immunostained human foreskin fibroblast cells, where the nanoparticles were broken down and the *Chlamydia* antigens displayed on the cell surface [66].

Recombinant GVNPs have also been produced displaying fragments of the *Salmonella enterica* serovar Typhi SopB protein and a *Plasmodium falciparum* enolase [40, 43–45]. Current vaccines for *Salmonella enterica* serovar Typhi use the Vi capsular polysaccharide subunit or live-attenuated vaccines [70]. However, the effectiveness of these vaccines is reduced by the need for multiple doses and there are also stability issues [70]. GVNPs were developed as an alternative *Salmonella enterica* vaccine candidate, with fragments of the secreted effector protein, SopB, fused to GvpC [40]. The *sopB* gene fragments were codon-optimized for expression in *Halobacterium* and the protein conformation on the GVNP was tested using antigen-specific sera [40]. For the first time using a recombinant GVNP vaccine, a live challenge was administered whereby mice were exposed to 10^7^ virulent *Salmonella* after the mice had been immunized and received a booster of the GVNP SopB vaccine [40]. A significant immune response was observed with increases in IFN-γ, IL-2 and IL-9, along with a reduction in the bacterial load of mice exposed to the vaccine compared with the NRC-1 GVNP control [40, 43]. The bacterial counts from the spleen, liver and mesenteric lymph nodes of mice exposed to the GVNP-SopB vaccine were reduced by at least two orders of magnitude [40, 43].

In *P. falciparum* work carried out by Dutta *et al*. an enolase was used as an antigen, as this protein had been found to localize to the cell surface in multiple stages of the *P. falciparum* life cycle [71]. A 15 amino acid peptide from the enolase enzyme was cloned into the previously described GVNP display system and the recombinant GNVPs were used to immunize mice before they were challenged with the murine parasite, *Plasmodium yoelii* [44]. Mice that were not immunized or were immunized with wild-type GVNPs showed significantly higher levels of parasitaemia compared to those found with the recombinant GVNPs displaying the enolase fragment [44]. Survival of mice immunized with the recombinant GVNPs was also significantly increased compared to that of the control groups [44].

In a recent study, the ability of recombinant GVNPs to rescue mice from endotoxic shock was determined, with promising results [41]. This was assessed using GVNPs displaying a fragment of the murine bactericidal/permeability increasing protein (BPI), an endotoxin neutralizing molecule [41, 72]. The BPI protein has anti-inflammatory properties, as it prevents the interaction between lipopolysaccharides of Gram-negative bacteria and Toll-like receptor 4 [41, 72]. This study utilized a new GVNP expression system that allows for the expression of GvpC with the insert of interest on a much smaller plasmid that does not contain the entire *Halobacterium* gas vesicle cluster, and is expressed in a *gvpC*-negative strain rather than a strain deleted for the entire gas vesicle cluster [73, 74]. The recombinant BPI–GVNPs displayed antibacterial activity, killing 50–75 % of *E. coli* and *S. typhi* cells when incubated together, and scanning electron microscopy showed evidence of bacterial cell lysis and membrane perturbations [41]. These BPI–GVNPs also elicited *in vivo* anti-inflammatory effects, with 100 % of mice injected in the footpad with BPI–GVNPs 1 h before challenge with bacterial lipopolysaccharide surviving for at least 5 days. In contrast, mice injected with wild-type GVNPs died within 7 h post-challenge [41]. The route of injection was important to the effect, however, with mice injected subcutaneously showing no difference in survival and mice injected intraperitoneally with BPI–GVNPs only showing an increased survival time of 24 h [41]. It was suggested that these results may be due to differences in absorption rates of the particles into the bloodstream [41, 75].

The *Halobacterium* sp. NRC-1 gas vesicles have shown great potential as a means to deliver antigens in vaccines. This system has been used to display epitopes from a diverse panel of pathogens and appears to be flexible in terms of the insertions tolerated in the GvpC protein, allowing for GvpC to retain functionality whilst antigens display the correct conformation. Tests performed *in vivo* have shown increased survival and reduction of disease in both *S. typhi* and *P. falciparum* infection models [40, 43, 44]. Most recently, this system has also been used to deliver an endotoxin-neutralizing protein that increased mouse survival in an endotoxic shock model [41].

The future usefulness of this system has been greatly improved by the development of a more efficient method for creating recombinant GVNPs [73]. This will enable a wide range of potential vaccine candidates to be investigated. The potential for GVNPs to be engineered to display multiple proteins together on the same particle is being investigated and would likely have a range of biotechnological applications [33]. Although these GVNPs are incredibly useful tools, unanswered questions still remain regarding basic aspects of how they are generated and organized within the cell [1, 76, 77]. Further work is needed to determine the roles of many of the proteins involved in the production and degradation of GVNPs and the exact method for how gas vesicles mature from bicones to cylinders is not well understood. The conformation of key proteins when displaying the antigens described above also remains unknown. Investigations into the optimal delivery route for these GVNPs are ongoing, with one study investigating the use of micro-needles to enhance skin permeation of the particles, with a view to developing them as a drug delivery system [78].

### Gas vesicles as contrast agents

There has long been interest in developing reporter genes that respond to magnetic resonance, analogous to genetically encoded optical reporters including the green fluorescent protein [79]. Due to their stable and rigid nature, gas vesicles have recently been developed as such a system for use in MRI [30, 34]. Current contrast agents for MRI and ultrasound are conventionally made from lipid or protein-stabilized gas microbubbles [34]. These microbubbles have limitations, as pressure gradients may lead to bubbles larger than 1 µm, which can result in fragmentation and subsequent escape of gas from the microbubbles [34, 80, 81]. This limits the usefulness of contrast agents when imaging vascular structures, as the microbubbles are unable to pass through the endothelium [80]. Ultrasound and MRI are capable of visualizing deep tissues within animal models and for human applications, but have few molecular reporters compared to optical imaging [82–84].

To test the potential to use gas vesicles as nanoscale ultrasonic molecular reporters, Shapiro and colleagues purified gas vesicles from *Anabaena flos-aquae* and *Halobacterium* sp. NRC-1, before imaging gel phantoms to see the contrast produced [34]. They also showed that, after gas vesicles have been collapsed by an increase in pressure, they are no longer able to produce any contrast [34]. This feature of gas vesicles can be exploited to generate subtraction images, whereby images are taken before and after gas vesicle collapse, to allow better contrast. A greater signal was also achieved when intact *A. flos-aquae* cells were imaged with gas vesicles contained inside [34]. Experiments were also carried out using a mouse model, where *Halobacterium* gas vesicles were injected into mice subcutaneously and intravenously, and could be detected via ultrasound [34].

Gas vesicles have also been used as reporters for hyperpolarized xenon MRI [85]. Hyperpolarized MRI is a more sensitive detection method that requires the presence of ^129^Xe, which can be detected in far lower concentrations than thermally polarized ^1^H, which is used in other reporter systems [85, 86]. Gas vesicles were purified and chemical exchange saturation transfer (CEST) was used to allow hyperpolarized xenon to diffuse into the vesicles. Gas vesicles could then be detected at concentrations of as low as 25 pM using this method [85]. The ability of GvpC to be engineered to produce gas vesicles with different collapse pressures was also investigated in order to create a range of gas vesicles with different acoustic properties that further enhance the range of ultrasound responses that could be realized [87]. Recombinant GvpC variants were expressed in *E. coli* and added to prepurified gas vesicles from *A. flos-aquae* that had their native GvpC proteins removed by treatment with 6 M urea, leaving only the GvpA shell intact [87]. These gas vesicles with different GvpC variants showed different acoustic collapse curves that could be distinguished from each other through imaging at different pressures [87].

The acoustic behaviour of *Halobacterium* sp. NRC-1 gas vesicles has been studied in greater detail to determine their suitability as contrast agents [88, 89]. These gas vesicles have different properties compared to those of *A. flos-aquae*; they are lemon-shaped rather than cylindrical and are wider than their cyanobacterial counterparts [1, 3, 88]. The acoustic collapse pressure of *Halobacterium* gas vesicles was determined at different frequencies, and was found to increase upon exposure to ultrasound to 620–694 kPa at 27.5 MHz from 522 to 576 kPa at 12.5 MHz [88, 89]. Finite element modelling simulations predicted that the collapse pressure is in part determined by the gas permeability of the gas vesicle shell [88]. Gas vesicles produce a strong second harmonic and behave nonlinearly at acoustic pressures above and below the critical collapse pressure [88, 90]. This indicates that gas vesicles can be detected using previously established methods used to detect microbubbles [89]. Similar experiments were also carried out using *A. flos-aquae* gas vesicles, either in their native form or with the GvpC shell removed, which created a greater harmonic response [87, 91].

There are detectable differences in the acoustic properties of gas vesicles isolated from different species [30, 34, 85]. This opens up the prospect of multiplexing, whereby different populations of gas vesicles can be visualized in the same sample through serial collapse imaging [30, 34]. Gas vesicles have been isolated from three different systems, *A. flos-aquae*, *Halobacterium* sp. NRC-1 and *E. coli* heterologously expressing gas vesicles from *Bacillus megaterium* [30, 34, 85]. Each system required different culturing and purification conditions, and gas vesicles could be purified from *E. coli* cells much more quickly than from cyanobacteria or haloarchaea (which can take 2–3 weeks to produce gas vesicles in sufficient quantities) [30]. These gas vesicles have been further manipulated to non-invasively image bacteria, producing them heterologously [92]. *E. coli* cells were transformed with a set of acoustic reporter genes (ARGs) from *A. flos-aquae* (the structural genes *gvpA* and *gvpC*) and *B. megaterium* (the accessory genes *gvpR-gvpU*) under an IPTG-inducible promoter [92]. *E. coli* cells making these combination gas vesicles produced greater ultrasound contrast compared to GFP-producing controls and were detectable at a concentration of 5×10^7^ cells ml^−1^ [92]. By varying the nature of the *gvpC* gene used, gas vesicles with differing collapse pressures were created and could be used to distinguish between populations [92]. Using a probiotic strain of *E. coli *, Nissle 1917, transformed with the acoustic reporter genes, the gastrointestinal tract of mice could be imaged using ultrasound, a location that is difficult to image by optical techniques [92].

A recent report has described how mammalian cells have been engineered to produce gas vesicles in a gene expression-dependent manner [93]. This system allows for the first time a link between the expression of genes in mammalian cells and an acoustic reporter that can be visualized by ultrasound [92, 93]. This work used codon-optimized genes from *B. megaterium* that were integrated into genome of HEK293T cells under a doxycycline-inducible promoter, with electron microscopy used to show successful gas vesicle production [93]. These gas vesicle-producing HEK cells were then used to create tumours in immunocompromised mice, which were treated with doxycycline to promote gas vesicle expression [93]. Using the previously described feature of gas vesicle collapse above certain ultrasound pressure thresholds, it was shown that only cells in the periphery of the tumour that were exposed to blood vessels produced gas vesicles, while those in the core of the tumour did not [34, 87, 92, 93]. This is an exciting step forward in the use of gas vesicles as imaging tools, but the authors note that there is still work to be done in condensing the constructs necessary for gas vesicle expression in mammalian cells to increase the utility of this reporter system [93].

The use of gas vesicles as biomolecular contrast agents has developed rapidly in recent years, with promising results shown *in vivo* and *in vitro* [34, 92]. Detailed modelling and experimental analysis of the acoustic behaviour of these gas vesicles has helped to develop a system that is functional with gas vesicles from different host organisms and that can be expressed heterologously [88, 89, 91, 92]. This system is still being optimized, with new applications currently in development, including the expression of gas vesicles in mammalian cells, the use of gas vesicles to image neural activity and the use of gas vesicles as optical coherence tomography contrast agents [92, 93]. Gas vesicles and targeted ultrasound have also been proposed as a means to deliver therapeutics through the selective destruction of engineered bacteria [94].

### Targeted collapse of gas vesicles as a biocontrol mechanism

Cyanobacteria are well known for their ability to grow in large blooms in the surface layers of standing water, often in lakes or reservoirs [95]. These blooms can be harmful to humans and animals that come into contact with, or consume, water contaminated with cyanotoxins [95]. Artificial mixing has been used as a method to control cyanobacterial blooms, as it eliminates stratification of the water, and can therefore change the dominant species by hampering the growth of cyanobacteria while favouring diatom growth [96, 97]. One feature that has been associated with the success of bloom-forming cyanobacteria is the ability to synthesize gas vesicles as a buoyancy aid [3, 98, 99]. The use of ultrasound to collapse gas vesicles has been proposed as a method to control cyanobacterial blooms [100]. The extent of the damage caused to cyanobacteria by ultrasound treatment is dependent on a range of factors, including the intensity and duration of the ultrasound and the frequency used [101]. An advantage of the sonication method is that it is more environmentally friendly than other methods to control blooms, such as the use of chemical algicides [101].

In an experimental setting, 3 s exposure to a 28 kHz frequency caused settling of 80 % of a culture of cyanobacteria; when assessed by transmission electron microscopy it was confirmed that the gas vesicles had collapsed following sonication [100]. A similar study found that growth of the gas vesicle-producing *Microcystis aeruginosa* was severely inhibited by the application of 1.7 MHz ultrasound, whilst the growth of *Synecochoccus* PCC7942 (which does not produce gas vesicles) was not affected [102]. Similarly, exposure to ultrasound of 1.7 MHz was sufficient to inhibit the growth of *Spirulina* (* Arthrospira *) *platensis*, a cyanobacterium that is not normally associated with bloom formation but does produce gas vesicles [103]. In pond and lake studies, the application of ultrasound and water pumps has been sufficient to control cyanobacterial levels without causing any death of larger organisms, such as fish and insects [104, 105]. However, further investigation may be necessary to determine that there have been no negative impacts short of death [104]. These outcomes were, in part, affected by the collapse of gas vesicles, but gas vesicle collapse and subsequent sedimentation of the cells does not necessarily lead to cell death and *de novo* biogenesis of gas vesicles can occur over time [104–106]. One concern surrounding the use of ultrasound to control cyanobacterial blooms is that, in addition to collapsing gas vesicles, ultrasound can also cause disruption of cell membranes and might lead to release of cyanotoxins, such as microcystins [101, 107]. Although ultrasound has been shown to also break down some microcystins, the effect of ultrasound frequency on cell integrity would be an important parameter to consider and so would need to be monitored to avoid further contamination of water [107, 108].

## Conclusions

Microbial gas vesicles show great promise as tools for various biotechnological, medical and environmental applications. They have utility for the engineering of better vaccines and the generation of novel MRI contrast agents, and can be exploited in the control of cyanobacterial blooms. Gas vesicles are stable structures and this is one of many features that make them attractive as exploitable biotechnological systems. If we are to benefit fully from the potential use of gas vesicles, the fundamental structural biology and molecular biology of vesicle morphogenesis and regulation require more intense study. Nevertheless, progress thus far has been exciting and so it seems highly likely that future applications of bacterial and archaeal gas vesicles in biotechnology, medicine and the environment hold substantial promise.
